# Youth and caregiver asthma functioning and quality of life throughout the COVID-19 pandemic

**DOI:** 10.3389/falgy.2023.1268112

**Published:** 2023-09-04

**Authors:** Manuela Sinisterra, Rachel Sweenie, Dima Ezmigna, David A. Fedele

**Affiliations:** ^1^ Department of Clinical and Health Psychology, University of Florida, Gainesville, FL, United States; ^2^ Division of Behavioral Medicine and Clinical Psychology, Cincinnati Children’s Hospital Medical Center, Cincinnati, OH, United States; ^3^ Department of Pediatrics, University of Florida, Gainesville, FL, United States

**Keywords:** pediatrics, quality of life, COVID-19, asthma control, asthma exacerbation

## Abstract

**Objectives:**

The COVID-19 pandemic resulted in daily functioning changes for many families. Adjustments in daily functioning may have impacted asthma management and subsequent morbidity. The current study seeks to build upon extant literature by exploring differences in youth asthma exacerbations and control, as well as youth and caregiver asthma-related quality of life (ArQOL) throughout COVID-19 transitional points.

**Methods:**

Ninety-three youth (9–17 years old) with asthma and their caregivers completed measures of demographic/medical information, asthma control, and ArQOL. Participants were recruited between January 2020 and October 2021 via their medical appointments and a hospital registry. We conducted Kruskal–Wallis *H*-tests to examine differences in youth asthma exacerbations (measured by short-acting beta agonist use), asthma control, and ArQOL, as well as caregiver ArQOL, across phases of the COVID-19 pandemic.

**Results:**

Asthma exacerbations were higher prior to the onset of the pandemic compared to “during lockdown” and “post-lockdown,” H(2) = 7.31, *p *< .05. Youth's asthma control was lower prior to the onset of the pandemic compared to youth enrolled “post-lockdown,” H(2) = 7.04, *p *< .05. There were no differences in youth ArQOL across the duration of the pandemic. Caregiver ArQOL was significantly higher in the “post-lockdown,” period, compared to caregivers enrolled prior to the pandemic onset, H(2) = 9.86, *p *< .01.

**Conclusion:**

Youth and caregiver asthma functioning improved following the onset of the pandemic. These findings build upon existing literature to highlight higher ArQOL in caregivers following the pandemic onset, likely related to improvements in youth asthma control and morbidity. Future research should explore trajectories of asthma and psychosocial functioning throughout the pandemic for families.

## Introduction

Asthma affects lung functioning by reducing airway diameter, which decreases airflow and causes potentially life-threatening symptoms (1). Respiratory viruses are a common asthma trigger. Thus, following the onset of the coronavirus disease (COVID-19) pandemic in 2020, regulatory guidelines indicated that individuals with asthma were at higher risk for complications related to COVID-19 and should take additional precautions to prevent transmission of the disease.

Several studies have since provided additional clarity about asthma risk, management, and outcomes in the context of the pandemic. In fact, research has found health-related improvements (i.e., morbidity, adherence) in individuals with asthma throughout the course of the pandemic. Overall, asthma control improved for many children and adolescents throughout the global pandemic (2). Hurst et al. (3) found that the frequency of pulmonary exacerbations requiring treatment with systemic steroids decreased during the pandemic. Simoneau et al. (4) also found that the percentage of youth asthma exacerbations requiring admission to the hospital was significantly lower in 2020 compared with 2019, and the frequency and proportion of emergency department (ED) visits for youth in 2020 was significantly lower than in 2018 and 2019. Arsenault et al. (5) further corroborated findings that youth ED visits significantly decreased in the first year following the pandemic onset. As a result of evidence found throughout the course of the pandemic, the American College of Allergy, Asthma, and Immunology (ACAAI) released statements confirming little to no increased risk of COVID-19 disease severity in individuals with asthma (6).

Many have speculated about the potential drivers of observed improvements in youth asthma functioning throughout the course of the pandemic. Hypothesized contributors to improvements include increased adherence to asthma medications, avoidance of the healthcare setting due to fear of contracting COVID-19, improved air quality due to school closures and “work-from-home” regulations, decreased participation in physical activity outside of the home, decreased exposure to asthma triggers, and decreased potential for viral exposure due to nationwide lockdowns (2, 3, 7). Additionally, families may have engaged in preventative measures for asthma management following the onset of the pandemic (i.e., refilling prescriptions, coordinating telehealth specialty care appointments) to avoid lapses in care during the healthcare state of emergency. Finally, families may have experienced increased vigilance to asthma management and overall health care, because of fear or anxiety around contracting COVID-19.

Although it is clear that there have been positive asthma-related morbidity and healthcare utilization outcomes in the context of the pandemic, less is known about the relationship between pandemic onset and youth and caregiver psychosocial functioning. Specifically, less attention has been given to highlighting whether the pandemic is related to any changes in health-related quality of life for youth or their caregivers. This area is critical to understand in the context of the pandemic, given established associations between health-related quality of life and asthma morbidity for youth (8–12), as well as other psychosocial outcomes for families (13–16).

The current study builds on burgeoning research on youth asthma functioning throughout the pandemic by describing differences in youth asthma exacerbations and asthma control throughout transitional points in the pandemic. Given previous findings indicating improvements in asthma management throughout the pandemic (5, 4), it was hypothesized that youth use of short-acting beta agonists (i.e., as needed rescue medications like albuterol taken in response to asthma symptoms) would be lowest during the pandemic lockdown period compared to pre-pandemic and post-lockdown. It was also hypothesized, in accordance with prior research (2), that youth asthma control would be highest during lockdown compared to pre-pandemic and post-lockdown. Additionally, we aimed to expand upon the current evidence base by elucidating differences in both youth and caregiver asthma-related quality of life across transitional points of the pandemic (i.e., pre-pandemic, during lockdown, and post-lockdown). We hypothesized that both youth and caregivers would report higher asthma-related quality of life during lockdown compared to pre-pandemic and post-lockdown, given related improvements in asthma symptoms and management.

## Methods

### Participants

This study was initially designed prior to the pandemic onset to examine associations between psychosocial functioning and asthma morbidity. Youth (ages 9–17) were eligible to participate if they had a physician-verified diagnosis of current asthma, were accompanied to their study visit by a parent or legal guardian who spent at least 3 days per week caring for them, and were able to speak and read in English (due to the availability of some questionnaires in English only). Caregivers had to be at least 18 years old and able to speak and read in English to participate. Families were excluded from participation if youth had a comorbid chronic health condition that might impact lung functioning (e.g., cystic fibrosis) or if youth or their caregivers had a significant cognitive impairment or developmental delay that might interfere with study completion (e.g., caregiver reported that their child would have difficulty completing questionnaires independently).

### Procedure

For participants recruited from outpatient clinics, study staff, with the help of the pulmonary medical team, identified potentially eligible participants by reviewing upcoming in-person and telehealth clinic appointments in the electronic medical record (EMR) system. During lockdown, we also requested a registry of patients with asthma who consented to be contacted for research opportunities; trained research assistants called eligible families to assess interest and screen for inclusion/exclusion. If a youth-caregiver dyad expressed interest in participating in the study following an initial introduction, a study staff member screened for eligiblity following the completion of youths’ clinic visit or over the phone. Families who met all inclusion and no exclusion criteria were invited to participate. Study staff then obtained consent/assent and administered questionnaires via Research Electronic Data Capture (REDCap) tools hosted at the principal investigator's institution. REDCap is a secure, web-based software platform designed to support data capture for research studies (17).

Recruitment and data collection began in January 2020, but study activities paused from mid-March through mid-April 2020 due to the university-wide stoppage of all in-person research at the start of the COVID-19 pandemic and while we transitioned to virtual study procedures. To accommodate for the transition away from in-person clinic visits and research, the study team began conducting study activities remotely (i.e., by Zoom videoconferencing, telephone, or mail). By June 2021, study recruitment and data collection returned to a primarily in-person format. The study protocol and all modifications made in response to the COVID-19 pandemic were approved by the local institutional review board. All eligible participants were invited to participate throughout the study periods; participation rates varied slightly, in that 34% of eligible participants recruited from in-person clinic visits enrolled in the study vs. 26% of all eligible participants recruited from the registry enrolled in the study.

### Measures

#### Sociodemographic and medical information

Caregivers reported their age, race, ethnicity, education and income, as well as the youth's age, race, and ethnicity. We used standardized items to collect education and income information from the MacArthur Research Network on Socioeconomic Status and Health (18). Caregivers also reported the number of youth's ED visits, hospitalizations, and missed school days in the past 12 months. Youth short-acting beta agonist use (in an average week) and oral steroid bursts (over the past 12 months) was also collected via caregiver-report using Likert scale questions based on NAEPP guidelines (8) and physician recommendation. Caregivers also reported their child's asthma severity, from very mild to very severe. Youths’ ED visits, hospitalizations, and asthma severity were corroborated by medical chart review.

#### Enrollment throughout COVID-19 pandemic

As noted above, recruitment of participants and data collection began prior to the onset of the COVID-19 pandemic and continued through October 2021. In order to elucidate on differences in variables of interest throughout the course of the pandemic, three distinct transitional time points were identified: pre-pandemic period (January 1 2020–March 15 2020), initial pandemic lockdown period (March 16 2020–August 17 2020), and post-lockdown period (September 17 2020–October 4 2021). Individual periods were determined in accordance with restrictions and regulations enacted by the institution's corresponding state legislature and aligned with distinct time points highlighted in extant literature (19).

#### Asthma control

Youth ages 9–11 and their caregivers completed the Childhood Asthma Control Test [C-ACT; (20)], and youth ages 12–17 completed the Asthma Control Test [ACT; (21)] independently. Both measures assess asthma symptoms, rescue medication use, and functional impairment over the past 4 weeks. Scores range from 0 to 27 (C-ACT) or 5–25 (ACT), with higher scores indicating better asthma control; scores ≤19 indicate poor control. The C-ACT and ACT have demonstrated good construct validity and internal consistency [*α* = .79 and .84, respectively; (20, 21)]. The C-ACT and ACT both demonstrated acceptable internal consistency (*α* = 0.73 and 0.78, respectively) in our sample.

#### Asthma-related quality of life

Caregivers completed the Pediatric Asthma Caregiver's Quality of Life Questionnaire [PACQLQ; (22)], a 13-item measure assessing two domains of quality of life: activity limitations (i.e., impact of their child's asthma on daily activities) and emotion function (i.e., as impacted by their child's asthma). Youth completed the Pediatric Asthma Quality of Life Questionnaire [PAQLQ; (23)], which measures impairment across domains of asthma symptoms, activity limitations (i.e., perceived impact of their asthma on daily activities), and emotional function (as impacted by their asthma) in the past 2 weeks. Responses for both the PACQLQ and PAQLQ are rated on a 7-point scale that ranges from “1 = Severe Impairment” to “7 = No Impairment.” Both subscale and summary scores were calculated. The PACQLQ and PAQLQ demonstrated excellent internal consistency (*α* = 0.90 and 0.95, respectively) in our sample.

### Overview of statistical analyses

All analyses were conducted using IBM SPSS version 27.0. Preliminary analyses included examining the distribution and normality of variables, as well as data missingness. The total number of participants per aim varied slightly due to missing asthma control questionnaire data from one participant. Pairwise deletion was used for this case and no additional data were missing. Youth asthma control, and youth and caregiver asthma-related quality of life scores were all significantly negatively skewed (Shapiro Wilke's *p *< 0.05). Blom transformations were conducted to attempt to correct negative skew, but variables remained significantly skewed. Therefore, the original, untransformed variables were utilized in non-parametric analyses. Data were also examined for associations between demographic variables (e.g., age, race and ethnicity, income) and outcome variables of interest.

To examine differences in youth use of short-acting beta agonists, asthma control, and asthma-related quality of life, as well as caregiver asthma-related quality of life, across phases of the COVID-19 pandemic, Kruskal-Wallis *H*-tests were conducted. The effect size of potential group differences was interpreted using eta-squared (*η*^2^), where *η*^2^ = 0.01 indicates a small effect, *η*^2^ = 0.06 indicates a medium effect, and *η*^2^ = 0.14 indicates a large effect (24).

## Results

### Enrollment throughout the COVID-19 pandemic

We recruited 23 families from their outpatient pulmonary clinic appointments prior to March 2020; we categorized these families as being enrolled *pre-pandemic*. We enrolled 18 families, using a remote protocol, throughout the initial pandemic lockdown period (mid-March 2020–August 2020), prior to the lifting of quarantine restrictions by state legislature; we categorized these families as being enrolled *during lockdown*. We enrolled 52 families, using a hybrid enrollment model of outpatient clinic appointments and a remote protocol, following the lifting of quarantine restrictions by the state legislature (mid-September 2020); we categorized these families as being enrolled *post-lockdown*. There were no differences in demographic characteristics between enrollment time points.

### Participants

Ninety-three dyads participated. Mean youth age was 12.6 ± 2.4 years. Fifty-four percent of youth were male and 46% were female. Youth racial identity was determined via caregiver report and included 48% White youth, 37% Black or African American youth, 14% multi-racial youth, and 1% Asian youth. Ninety percent of youth were of non-Hispanic or Latinx ethnic background, per caregiver-report. Less than one-quarter of youth (23%) had intermittent or exercise-induced asthma, less than one-third (27%) had mild persistent asthma, about a third (31%) had moderate persistent asthma, and 12% of youth had severe persistent asthma. See Table 1 for additional youth demographic and medical information.

**Table 1:** Youth demographic and asthma characteristics.

	*M (SD)*	Range
Age (years)	12.57 (2.43)	9–17
Emergency department visits (past year)	.76 (1.34)	0–6
Hospitalizations (past year)	.34 (1.18)	0–10
Missed school days (past year)	1.66 (3.66)	0–24
	*Mdn*	Range
Asthma control (as measured by Asthma Control Test)	23	10–27
	*n*	%
Sex		
Male	50	53.8
Female	43	46.2
Race		
Asian	1	1.1
Black or African American	34	36.6
White	45	48.4
Multiracial	12	13.9
Not reported	1	1.1
Ethnicity		
Non-Hispanic/Latinx	84	90.3
Hispanic/Latinx	8	8.6
Not reported	1	1.1
Asthma subtype		
Exercised-induced	2	2.2
Intermittent	19	20.4
Mild persistent	25	26.9
Moderate persistent	29	31.2
Severe persistent	11	11.8
Not specified	7	7.6
Prescribed daily controller medication	69	74.2
Use of short-acting beta agonists (past month)		
Not used	39	41.9
1–2 days per week	28	30.1
3–4 days per week	10	10.8
5–6 days per week	6	6.5
Every day of the week	8	8.6
Don't know	2	2.2
Oral steroid bursts (past year)		
None	33	35.5
One	28	30.1
Two	12	12.9
Three	11	11.8
Four or more	5	5.4
Don't know	4	4.3

Mean caregiver age was 41.3 ± 8.2 years. Most caregivers identified as female (93%) and biological mothers (85%). Fifty-three percent of caregivers reported their race as White, while 37% identified as Black or African American, 7% as multi-racial, and 4% as Asian. Most caregivers (90%) identified as non-Hispanic or Latinx. About 26% of families who reported on annual income (ten caregivers chose not to report annual income), lived below the poverty line according to federal poverty guidelines (25). Approximately one-third (33%) of caregivers had a bachelor's degree or higher and about half (58%) were married at the time of assessment. See Table 2 for caregiver demographic information.

**Table 2:** Caregiver demographic characteristics.

	*M (SD)*	Range
Age (years)	41.26 (8.22)	28–72
	*n*	%
Sex		
Female	86	92.5
Male	7	7.5
Relationship to youth		
Biological mother	79	84.9
Biological father	6	6.5
Step, adoptive, or foster mother	3	3.2
Step, adoptive, or foster father	1	1.1
Grandmother	3	3.2
Other	1	1.1
Race		
Asian	4	4.3
Black or African American	34	36.6
White	49	52.7
Multiracial	6	6.5
Ethnicity		
Non-Hispanic/Latinx	84	90.3
Hispanic/Latinx	8	8.6
Not reported	1	1.1
Marital status		
Currently married	54	58.1
Single, divorced	16	17.2
Single, never married	18	19.4
Single, co-habiting	3	3.2
Single, widowed	2	2.2
Below federal poverty line ($26,500 for family of 4^a^)	24	25.8
Education		
Less than high school	3	3.2
Other (e.g., certificate)	7	7.5
High school diploma or equivalency	35	37.6
Associate degree	17	18.3
Bachelor's degree	19	20.4
Master's degree	5	5.4
Doctorate	6	6.5
Professional (MD, JD, DDS, etc.)	1	1.1

^a^ Computations for the 2021 annual update of the health and human services poverty guide.

### Use of short-acting beta agonists

Approximately 40% (*n* = 39) of youth did not use short-acting beta agonists in the past month, 30% (*n *= 28) used the medications 1–2 days per week, 17% (*n *= 16) used medication 3–6 days per week, and less than 10% (*n *= 8) of youth used them every day of the week. Two caregivers were unsure of the frequency of their child's short-acting beta agonist use.

Youth's frequency of use of short-acting beta agonists varied significantly between the pandemic time points [H(2) = 7.32, *p *< .05, *η*^2^ = .079]. Pairwise comparisons indicated that youth enrolled pre-pandemic had significantly greater weekly use of short-acting beta agonists than did youth enrolled during lockdown (*p* < .05); there was also a trend in the same direction between youth enrolled pre-pandemic and youth enrolled in the post-lockdown period (*p* = .06).

### Asthma control

When examining scores for the full sample, most youth reported that their asthma was well controlled (*Mdn *= 23); 18% of youth reported having poorly controlled asthma (i.e., C-ACT or ACT score <20). Importantly, youth's asthma control differed significantly between time points of the pandemic [H(2) = 7.03, *p* < .05, *η*^2^ = .077]. Asthma control was significantly lower in youth enrolled enrolled pre-pandemic (*Mdn *= 19), compared to youth enrolled during lockdown (*Mdn *= 24) or youth enrolled in the post-lockdown period (*Mdn *= 23; all *ps* < .05).

### Asthma-related quality of life

#### Youth

In the full sample, median asthma-related quality of life for youth was 6.0. Youth quality of life in domains of activity limitations, symptoms, and emotion function were similar, as median scores for each subscale for the full sample were 6.4, 6.0, and 6.1, respectively. No significant differences between subscale scores were observed. There were also no significant differences in youth asthma-related quality of life between pandemic time points. Quality of life scores were reported as equally high for youth enrolled prior to the start of the pandemic (*Mdn *= 6.1), during lockdown (*Mdn *= 6.4), and in the post-lockdown period (*Mdn *= 5.9).

#### Caregivers

**Figure 1:**
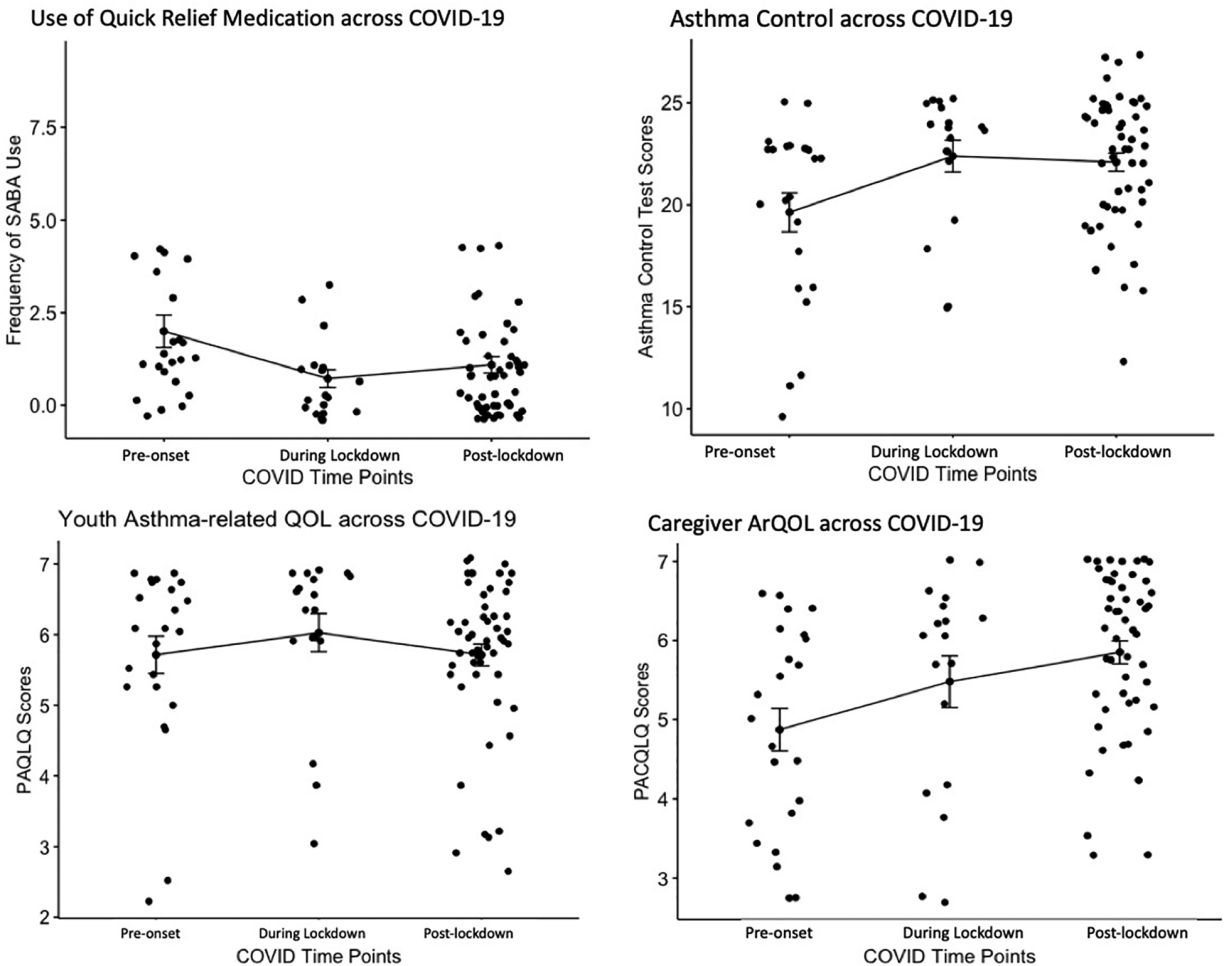
Comparing outcomes pre-pandemic, during lockdown, and post-lockdown. SABA, short-acting beta agonist; PAQLQ, pediatric asthma quality of life questionnaire; PACQLQ, pediatric asthma caregiver's quality of life questionnaire; ArQOL, asthma-related quality of life.

In the full sample, median asthma-related quality of life for caregivers in this sample was 5.8. Median scores were significantly higher for the full sample (i.e., higher asthma-related quality of life) in the domain of emotional function (*Mdn *= 6) than for the domain of activity limitations (*Mdn *= 5.7), *z* = −2.63, *p* < 0.01, *r* = 0.27. Unlike for youth, caregiver asthma-related quality of life varied significantly between pandemic time points [H(2) = 9.9, *p* < .01, *η*^2^ = .107]. Pairwise comparisons indicated that caregivers of youth enrolled pre-pandemic reported significantly lower asthma-related quality of life (*Mdn *= 5), compared to caregivers enrolled in the post-lockdown period (*Mdn *= 6.1). There were no significant differences in asthma-related quality of life between caregviers enrolled pre-pandemic and those enrolled during the lockdown period. See Figure 1 for a comparison of outcomes between pandemic time points.

## Discussion

This study aimed to characterize youth asthma exacerbations, control, and quality of life across three distinct phases of the COVID-19 pandemic, as well as to describe caregiver asthma-related quality of life in this context. Differences in youth and caregiver asthma functioning emerged based on time point during the pandemic, including “pre-pandemic,” “lockdown,” and “post-lockdown.” Broadly, medical and psychosocial information collected from participants in the “lockdown” and “post-lockdown” periods indicated better overall asthma functioning than that of their “pre-pandemic” counterparts. Specifically, youth enrolled prior to the onset of the pandemic reported more frequent weekly use of short-acting beta agonists, an indicator of asthma exacerbation, than youth enrolled during lockdown. Weekly use of short-acting beta agonists was also slightly lower for youth enrolled in the post-lockdown period, compared to youth enrolled prior to the onset of the pandemic. Youth enrolled before the pandemic onset also reported lower asthma control than those enrolled during lockdown or in the post-lockdown period. These findings are consistent with recent studies highlighting asthma outcomes for youth throughout the COVID-19 pandemic, which are most likely related to increased parental monitoring (26) and decreased exposure to asthma triggers (2, 3) throughout the course of the pandemic and into the post-lockdown period. Despite the lifting of stay-at-home orders and re-opening of schools, improvements in asthma morbidity may have persisted into the post-lockdown period due to behavioral changes to asthma management made during lockdown (i.e., parental monitoring continued with flexible work schedules). Further, it is possible that asthma exacerbations were lowest and asthma control was highest during the “lockdown” period due to increased vigilance around asthma management resulting from concerns of contracting COVID-19 or having limited access to hospital/emergency resources in the case of an exacerbation.

Interestingly, there were no differences in asthma-related quality of life for youth between distinct phases of the pandemic; ratings of youth asthma-related quality of life remained steadily high, on average, regardless of time of enrollment. This finding indicates that youth may not generally perceive significant day-to-day impairment from managing their asthma, and those sentiments did not appear to differ based on phase of the pandemic. Considered in the context of the ongoing youth mental health crisis that arose during COVID-19 lockdown, findings suggest that impairment from asthma may be a separate consideration, or that youth in this sample may have had fewer mental health struggles than the general population (27). In contrast to youth, caregiver asthma-related quality of life did differ across phases of the pandemic, wherein caregivers enrolled during lockdown and post-lockdown reported significantly greater quality of life than their “pre-pandemic” counterparts. Again, this is in contrast to current literature, which suggests that caregivers of children with chronic health conditions were vulnerable to worse well-being during the COVID-19 pandemic (28). Caregivers’ asthma-related quality of life while in lockdown and post-lockdown may have benefitted from their child's lower use of short-acting beta-agonists or greater asthma control, and thus contributing to caregivers feeling less impacted by their child's asthma. With the transition to school and work from home that occurred after the onset of the pandemic, with many employers continuing to support flexible work arrangments, many caregivers have also had increased opportunities to easily monitor their child's asthma symptoms and management (26); this may have led to lower perceived impacts of their child's asthma on their own day-to-day responsibilities and emotional functioning for some caregivers. Should that be the case, the findings support recommendations in the extant literature in favor of parent-child collaborative asthma management in order to support positive asthma-related quality of life for caregivers (29–32).

### Strengths and limitations

This study has several notable strenghts. First, the timing of this study offered a unique opportunity for us to examine differences in asthma morbidity and quality of life between distinct phases of the pandemic. In fact, this study is one of the first to examine caregiver-specific asthma functioning in the context of the pandemic. Participants in this study also represented a diverse population in terms of race and socioeconomic status, which increases the generalizability of our findings.

Findings should also be considered in the context of several limitations. Most data were collected following the onset of the COVID-19 pandemic, resulting in uneven sample sizes for analyses that limit the interpretation of findings. Sample sizes and skewed data required us to use non-parametric statistical analyses, which also limits interpretation. Families were primarily recruited through a pediatric asthma clinic or a registry of existing hospital patients. Thus, a large portion of the sample included families who were able to access subspecialty pediatric care; families whose asthma care may have been impacted by the pandemic (i.e., families who receive their asthma care through ED visits) or who faced other challenges to their asthma management or quality of life (e.g., lack of insurance coverage due to job loss, deaths of loved ones) may have not been as frequently captured in this study. Additionally, we did not collect information about participants’ reason for attending their medical appointment (i.e., routine visit, sick visit, prescription renewal). If participants were attending a subspecialty care visit because of an asthma exacerbation or other sick symptoms, it may have impacted their asthma control scores. Another important limitation is that youth and caregiver psychosocial functioning was only assessed within the context of asthma-related quality of life. The onset of the COVID-19 pandemic had several implications for families’ psychosocial functioning above and beyond asthma care (i.e., mood symptoms, stress). For example, caregivers may have experienced elevated symptoms of depression and anxiety in response to the pandemic onset, as well as stress associated with transitioning to home-based work and education (28, 33–35). These domains of functioning are also important to explore, as they likely contribute to youth and caregivers’ ability to manage asthma adequately.

### Future directions

It would be beneficial to assess trajectories of youth and caregiver asthma functioning during the pandemic, in addition to identifying isolated differences in functioning at distinct time points in the pandemic. This could be accomplished by examining data from larger samples in other studies that were underway at the onset of the pandemic and/or by extracting electronic medical record (EMR) data, which typically includes information on exacerbations and assessment of asthma control. For example, researchers could extract EMR data and assess asthma morbidity in the context of the pandemic within a sample of families seen in primary care clinics and EDs. This would enable identification of broader patterns in functioning in a larger sample over time, which would allow for more systematic assessment of the potential drivers of improvements in asthma morbidity and quality of life. Future research should also assess youth and caregiver mental health to determine how these relate to asthma management in the context of the COVID-19 pandemic. It is crucial to better understand trajectories of youth and caregiver mental health throughout the course of the pandemic, and subsequently understand how changes in functioning may have impacted asthma management, to inform clinical care. For example, regularly screening for mental health concerns like anxiety and depression and social determinant of health-related challenges that arose during the pandemic as well as embedding mental health providers (i.e., psychologists, social workers) in asthma clinics to address concerns may improve asthma management and clinical outcomes.

Additionally, it may be beneficial to further explore the current research questions utilizing qualitative methodologies, to better understand the potential drivers of improvements in youth asthma morbidity, as well as caregiver asthma-related quality of life. Youth and caregiver perspectives could help pinpoint targets for clinical intervention to improve pediatric asthma functioning above and beyond the context of the pandemic. In particular, it will be important to highlight caregiver feedback on contributors to improved asthma-related quality of life and integrate discussion of caregiver functioning into clinical care.

## Conclusion

This study characterized and compared youth asthma exacerbations and control as well as youth and caregivier asthma-related quality of life before the COVID-19 pandemic, during lockdown, and in the post-lockdown period. We found that, in general, short-acting beta agonist use was lowest and asthma control and caregiver quality of life were highest during the lockdown and post-lockdown periods, which is consistent with previous research suggesting an overall decrease in asthma morbidity during the COVID-19 pandemic. Our findings also highlight the importance of understanding caregivers’ psychosocial well-being in the context of pediatric asthma, particularly suggesting that caregiver quality of life may be an important clinical consideration. Future research should examine trajectories of asthma morbidity and psychosocial functioning pre-pandemic, during lockdown, and post-lockdown using larger samples from studies that occurred during the pandemic as well as EMR data.

## Data Availability

The datasets for this article are not publicly available due to concerns regarding participant/patient anonymity. Requests to access the datasets should be directed to the corresponding author.
